# Gene promoter DNA methylation patterns have a limited role in orchestrating transcriptional changes in the fetal liver in response to maternal folate depletion during pregnancy

**DOI:** 10.1002/mnfr.201600079

**Published:** 2016-06-06

**Authors:** Jill A. McKay, Michiel Adriaens, Chris T. Evelo, Dianne Ford, John C. Mathers

**Affiliations:** ^1^ Human Nutrition Research CentreInstitute of Health and Society, Newcastle UniversityNewcastle upon TyneUK; ^2^ Maastricht Centre for Systems Biology—MaCSBioMaastricht UniversityMaastrichtthe Netherlands; ^3^ Department of Bioinformatics—BiGCaTMaastricht UniversityMaastrichtthe Netherlands; ^4^ Human Nutrition Research CentreInstitute for Cell and Molecular BiosciencesNewcastle UniversityNewcastle upon TyneUK; ^5^ Human Nutrition Research CentreInstitute of Cellular MedicineNewcastle UniversityNewcastle upon TyneUK

**Keywords:** Development, DNA methylation, Folate, Gene expression, Programming

## Abstract

**Scope:**

Early‐life exposures are critical in fetal programming and may influence function and health in later life. Adequate maternal folate consumption during pregnancy is essential for healthy fetal development and long‐term offspring health. The mechanisms underlying fetal programming are poorly understood, but are likely to involve gene regulation. Epigenetic marks, including DNA methylation, regulate gene expression and are modifiable by folate supply. We observed transcriptional changes in fetal liver in response to maternal folate depletion and hypothesized that these changes are concomitant with altered gene promoter methylation.

**Methods and results:**

Female C57BL/6J mice were fed diets containing 2 or 0.4 mg folic acid/kg for 4 wk before mating and throughout pregnancy. At 17.5‐day gestation, genome‐wide gene expression and promoter methylation were measured by microarray analysis in male fetal livers. While 989 genes were differentially expressed, 333 promoters had altered methylation (247 hypermethylated, 86 hypomethylated) in response to maternal folate depletion. Only 16 genes had both expression and methylation changes. However, most methylation changes occurred in genomic regions neighboring expression changes.

**Conclusion:**

In response to maternal folate depletion, altered expression at the mRNA level was not associated with altered promoter methylation of the same gene in fetal liver.

**Abbreviations**
NTD: neural tube defect
PGE: positional gene enrichment
SAM: *S*‐adenosyl‐methionine

## 1 Introduction

The developmental origins of health and disease hypothesis suggests that exposures during early life are critical in altering the programming of a developing fetus, and that adverse exposures may predispose to the development of noncommunicable disease(s) in later life. Such adverse exposures include under‐ and overnutrition during fetal and neonatal development, which increase susceptibility to a wide range of adult diseases 1. Adequate maternal consumption of folate (one of the group of B vitamins) during pregnancy is essential to ensure healthy fetal development and, in particular, to protect against the development of neural tube defects (NTDs) 2, 3. Further epidemiological evidence suggests that folate supplementation during pregnancy may also reduce the risk of other congenital defects 4 and adverse pregnancy outcomes 5, as well enhancing neurodevelopment 6 and reducing the risk of severe language delay 7, autism 8, 9, and some cancers (leukemia 10, 11, 12, brain tumors 13, 14, and neuroblastoma 15) 16 in children. Moreover, folate deficiency during pregnancy in rodents can cause spontaneous abortion, teratogenic effects in offspring, reduced litter numbers, and altered body weight of offspring 17, 18, emphasizing the essentiality of sufficient maternal folate intake for successful pregnancy outcomes, normal fetal development, and long‐term offspring health.

The mechanisms underlying such fetal programming events are poorly understood, but are likely to involve changes in gene regulation. Given the critical importance of ensuring that cells express the appropriate consortium of genes to match their circumstances, it is not surprising that a wide range of mechanisms are employed in the regulation of gene expression, including mechanisms involved in transcription of genomic sequences to mRNA. In addition to specificity factors, repressors, transcription factors, activators, enhancers, and silencers, all of which influence gene transcription, epigenetic mechanisms provide a further‐integrated machinery for transcriptional control. Epigenetic marks, including DNA methylation and histone modifications, are copied from one cell generation to the next and these epigenetic marks and noncoding microRNAs (miRNA) work together as the “epigenome” to regulate gene expression 19, 20. Importantly, multiple environmental factors alter epigenetic patterns, and in particular DNA methylation, suggesting that epigenetic processes are a mediating mechanism by which environmental factors influence gene transcription 21 and cell function. Folate is central to one‐carbon metabolism and to the formation of the universal methyl donor *S*‐adenosyl‐methionine (SAM), which is critical for the methylation of biological molecules including DNA, lipids, and proteins. Indeed the influence of dietary folate intake on DNA methylation patterns has been reported widely in human 22, 23, 24, 25, 26 and animal studies 27, 28, 29, 30, 31, but few studies have examined the effects of this methylation change on gene transcription. To our knowledge, no study has investigated the impact of dietary folate alone on both gene expression and methylation at the genome‐wide level.

We have observed widespread transcriptional changes (555 upregulated and 434 downregulated genes) in the fetal livers of male C57BL/6J offspring at 17.5‐day gestation whose mothers were fed diets containing 0.4 mg folic acid/kg for 4 wk before mating and throughout pregnancy compared with controls (i.e., mothers fed 2 mg folic acid/kg; McKay et al., “Organ‐specific gene expression changes in the fetal liver and placenta in response to maternal folate depletion,” currently under review). Given its role in SAM production, we hypothesized that these transcriptional changes in response to folate intake may be due to altered epigenetic regulation of gene expression, more specifically, by the modification of DNA methylation patterns. To test this hypothesis, we investigated genome‐wide promoter methylation status in the fetal liver in response to maternal folate depletion. To facilitate the integration of transcriptomic and epigenomic datasets, methylation analysis was carried out in the same animals in which genome‐wide gene expression had been quantified previously.

## 2 Materials and methods

### 2.1 Animal husbandry and experimental diets

All animal procedures were approved by the Newcastle University Ethics Review Committee and the UK Home Office (project license number 60/3979) and have been described previously 29. Animals were housed in the Comparative Biology Centre, Newcastle University, at 20–22°C and with 12‐h light and dark cycles. Fresh water was available ad libitum. Female C57BL/6J mice were allocated at random to either a low‐folate (0.4 mg folic acid/kg diet) or normal‐folate diet (2 mg folic acid/kg diet; 6 g of allocated diet was offered to each mouse per day), and maintained on this diet for 4 wk prior to mating. Diet compositions were modified from AIN‐93G 32 and have been described previously 29. Mice were time mated, that is, a male was added to a cage containing two females overnight and removed the following morning. Pregnant females, identified by the presence of a vaginal plug, were recaged and offered 10 g/day of allocated diet throughout pregnancy. At 17.5‐day gestation, dams were killed for collection of blood and tissues.

### 2.2 Sample collection

Animals were anesthetized using gaseous isoflurane, and animals killed by cervical dislocation. The uterus, containing all fetuses and placentas, was removed and placed immediately in ice‐cold PBS. The liver of each fetus was removed, weighed, and snap frozen in liquid nitrogen and stored at –80°C.

### 2.3 RNA extraction, gene expression arrays, and validation using real‐time PCR

RNA extraction protocols, array hybridization, and subsequent data analyses have been described and data presented elsewhere (McKay et al., “Organ‐specific gene expression changes in the fetal liver and placenta in response to maternal folate depletion,” currently under review). Briefly RNA was extracted from fetal livers of males only using Tri‐reagent (Sigma‐Aldrich) and following the manufacturer's instructions. Genome‐wide transcript abundance was determined by ServiceXS (Plesmanlaan 1/D, 2333 BZ Leiden, the Netherlands) on the Affymetrix GeneChip platform with the NuGO mouse array (NuGO_Mm1a520177). This array comprises over 24 000 probe sets, covering the majority of established genes. All raw and processed microarray data have been deposited in the ArrayExpress database E‐MTAB‐3940.

### 2.4 DNA extraction

DNA was extracted from male fetal livers using Tri‐reagent (Sigma‐Aldrich) as per manufacturer's instructions. Briefly, 50 mg tissue was homogenized in 500 μL Tri‐reagent on ice. A further 500 μL tri‐reagent and 200 μL chloroform were added, the sample was mixed by inversion and incubated on ice for 5 min. Samples were centrifuged at 13 500 rpm for 15 min at 4°C. The upper aqueous phase was removed, and 300 μL 100% ethanol added to the lower phases, mixed by inversion, and incubated for 2 min at room temperature before centrifugation at 2000 × *g* for 5 min at 4^⁰^C. The supernatant was removed, and the pellet was washed twice, incubating at room temperature for 30 min in 1 mL of 0.1 M sodium citrate in 10% ethanol with periodic mixing, followed by centrifugation at 2000 × *g* for 5 min at 4°C. The DNA pellet was suspended in 1.5 mL 75 % ethanol for 20 min at room temperature with periodic mixing, followed by centrifugation at 2000 × *g* for 5 min at 4°C. Ethanol was removed and the pellet allowed to air dry for 3–5 min at room temperature. DNA was resuspended in 300 μL 8 M NaOH and purity and concentration were determined using a Nanodrop Spectrophotometer (ThermoScientific).

### 2.5 Methylated DNA immunoprecipitation and methylation array hybridization

**Figure 1:**
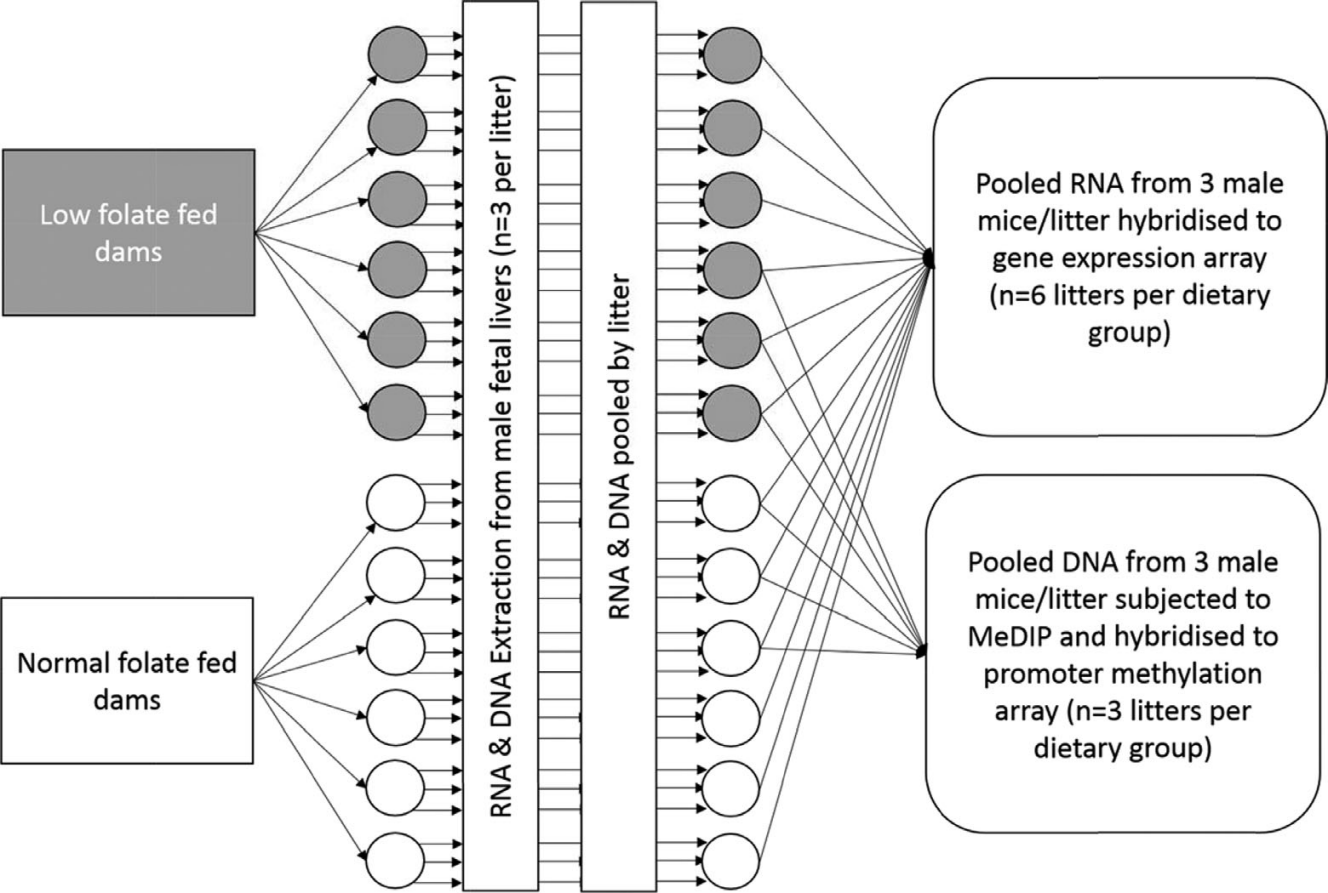
Schema depicting study design and sample selection for genome‐wide analyses.

The methylated DNA immunoprecipitation (MeDIP) protocol has been described in detail elsewhere 33. For six litters in which transcriptomic analysis had been carried out (*n* = 3 per dietary group), DNA was pooled for three male fetuses per litter (5 μg/fetus) prior to preparation for MeDIP (see Fig. 1 for overview of study design). Briefly, 10 μg of DNA was incubated at 37°C for 30 min with 20 μL A/T1 RNase (Fermentas) in a 500 μL volume. DNA was sonicated in cold water for 2 min at 20% pulse and 5 V of power output with an Ultrasonic Homogenizer 4710 Series (Cole‐Parmer Instrument). Extent of sonication was assessed by loading 15 μL sample on a 1% agarose gel with 0.4 μg/mL of ethidium bromide. Successfully fragmented DNA samples (200–1000 bp) were concentrated with silica columns (Zymo Research) to 50 μL in TE buffer. To assess the success of the immunoprecipitation, genomic DNA was spiked with positive (i.e., methylated) and negative (i.e., unmethylated) control PCR products derived from lambda phage DNA (described previously 33). Reaction mixtures were prepared by adding 40 ng of positive control and 40 ng negative control to 4.4 μg of sonicated DNA in a total volume of 495 μL of TE buffer. After 10 min of denaturation at 95°C, samples were cooled on ice for 10 min and one‐tenth of the reaction volume was stored as input at 4°C. Immunoprecipitation reactions were performed as described previously 33. PCR amplification of spiked positive and negative controls for both MeDIP and input samples was carried out to confirm methylated controls were present in both MeDIP and input samples, and that unmethylated amplicons were present only in input samples (see Lisanti et al. 33 for details of PCR, data not shown). Whole‐genome amplification of MeDIP and input samples was carried out using a WGA2 kit (Sigma) following the manufacturer's instructions, with 20 ng of template DNA and omitting the fragmentation step. Products of the WGA reactions were purified on silica columns (Qiagen) and elutions were performed with 50 μL water. Prior to array hybridization, quantitative PCR was carried out to confirm the enrichment of methylated DNA in MeDIP compared with corresponding input samples as described previously 33 (data not shown). Five micrograms of MeDIP and input DNA were sent to NimbleGen Roche for hybridization to two‐channel MM8_RefSeq_promoter methylation arrays.

All samples passed the quality control analysis performed in the arrayQualityMetrics package 34. Next, the raw DNA methylation data were normalized using T‐quantile normalization applied across samples and separately on the two channels 35 (corresponding to the MeDIP (Cy5, 635nm) and input DNA samples (Cy3, 532nm), respectively). Then, for each diet, enrichment scores were calculated, defined as the negative log_10_‐transformed *p*‐value obtained from a Kolmogorov–Smirnov sliding window approach. This approach assesses whether the probe intensities (defined as the log_2_‐transformed ratio between the channels, i.e., the MeDIP and input DNA intensities) observed in a genomic window (for all samples together; *n* = 3) are significantly higher than that expected from the complete intensity distribution of all probes. The genomic window size was set at 750 bp and only genomic windows with at least four probes were considered, in accordance with the manufacturer's guidelines. The average probe‐spacing for the array is 100 bp, yielding on average seven measurements per genomic window. Next, for each annotated promoter and for each diet only the genomic window with the highest enrichment score, in addition to the corresponding genomic window for the other diet, was considered for further analysis.

Finally, for each sample (*n* = 6) DNA methylation intensity values were calculated based on the mean probe intensity value in each considered genomic window. To assess differences in methylation intensity between diets (*n* = 3 for each diet), a linear modeling approach implementing heteroscedasticity‐consistent standard errors 36 was applied using the *lmtest* and *sandwich* packages in R. This yielded a differential methylation *p*‐value and a differential methylation fold change. Genes were considered to be significantly differentially methylated in response to low maternal folate intake if there was a significant (*p* < 0.05) fold change of at least ±1.2 fold. All raw and processed microarray data have been deposited in the ArrayExpress database E‐MTAB‐4013.

### 2.6 Gene ontology enrichment and pathway analysis

DAVID 37 was used to carry out Gene Ontology enrichment analysis and to investigate KEGG pathways affected by maternal folate depletion through changes in gene expression and DNA methylation. The threshold for significance for Gene Ontology enrichment analysis was set at *p* < 0.05 (corrected for multiple testing), and at *p* < 0.05 (uncorrected) for KEGG pathway enrichment analysis. Additional pathway analysis was carried out using PathVisio 38 3.2.0 and the curated pathway collection of WikiPathways 39 (download date: 01‐09‐2015), applying a significant (*p* < 0.05) fold change of at least ±1.2 fold, imposing a *Z*‐score of 1.9 for significance to filter for probable changed pathways

### 2.7 Positional gene enrichment (PGE) analysis

PGE analysis was carried out using the web‐based PGE tool (http://homes.esat.kuleuven.be/∼bioiuser/pge/index.php) 40 to locate overrepresented chromosomal regions for significantly upregulated, downregulated, hypermethylated, and hypomethyled genes in the fetal liver in response to low maternal folate intake.

This tool applies an algorithm using the hypergeometric distribution to test if a chromosomal region is enriched in a given set of genes. A region is determined to be pertinent if it contains at least two genes of interest, there is no smaller region containing the same genes of interest, there is no bigger region with more genes of interest and the same genes not of interest, there is no larger encompassing region with a higher percentage of genes of interest, there is no smaller encompassed region with a better *p*‐value, and it does not contain any region having less than expected genes of interest 40. Resultant data were mapped to chromosomal locations using Ensembl (http://www.ensembl.org).

## 3 Results

### 3.1 Influence of maternal folate intake during pregnancy on gene expression in the fetal liver

Changes in gene expression in fetal liver in response to maternal folate depletion have been described in detail elsewhere (McKay et al., “Organ‐specific gene expression changes in the fetal liver and placenta in response to maternal folate depletion,” currently under review). In summary, 989 genes were differentially expressed, of which 555 were upregulated and 434 downregulated, and 7.4% of differentially expressed genes coded for transcription factor proteins. Analysis of WikiPathways using PathVisio suggested changes in gene expression in response to maternal folate depletion may have influenced 13 biological pathways (“Striated Muscle Contraction, Adipogenesis genes, Fatty Acid Biosynthesis, Iron Homeostasis, TGF Beta Signaling Pathway, miR‐1 in cardiac development, One‐carbon metabolism and related pathways, EPO Receptor Signaling, Spinal Cord Injury, Alanine and aspartate metabolism, Methylation, and Osteoblast”; (McKay et al., “Organ‐specific gene expression changes in the fetal liver and placenta in response to maternal folate depletion,” currently under review).

### 3.2 Influence of maternal folate intake during pregnancy on DNA methylation in the fetal liver

**Figure 2:**
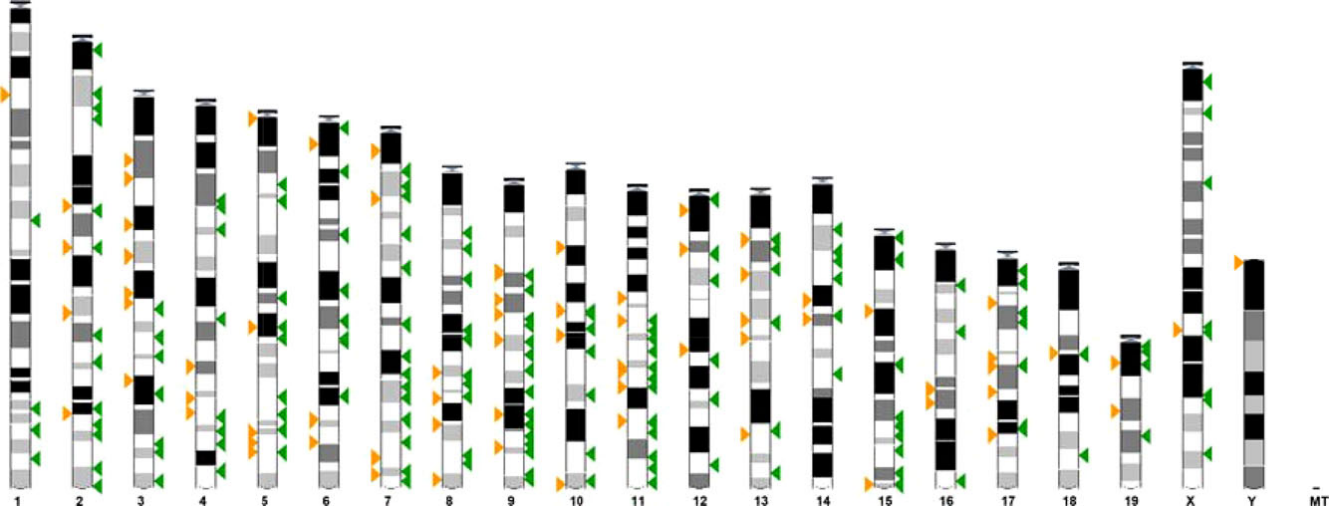
Chromosome locations of genes with methylation change in response to low maternal folate intake in the fetal liver. Green arrows represent hypermethyled genes, orange arrows represent hypomethylated genes.

In response to maternal folate depletion, 333 genes were differentially methylated in the fetal liver of which 247 were hypermethylated and 86 were hypomethylated (see Supporting Information Table 1 for full gene lists). Methylation changes were distributed across the genome rather than being localized to specific chromosomal regions (Fig. 2).

Pathway analysis using PathVisio found 12 WikiPathways in which a significant number of genes had altered methylation in the fetal liver in response to low maternal folate intake (Table 1). Additionally, KEGG pathway analysis revealed that inadequate maternal folate intake resulted in a significant number of genes in the “steroid hormone biosynthesis” pathway with altered methylation in the fetal liver (Table 2). However, Gene Ontology analysis found no statistically significant influence of the reported gene expression changes on biological processes.

**Table 1:** WikiPathways in which gene methylation was altered in the fetal liver in response to low maternal folate intake during pregnancy

Pathway	Number of genes altered on pathway	Number of genes measured on pathway	Total number of genes on pathway	Hypermethylated genes	Hypomethylated genes	Percentage affected	*Z*‐score	*p*‐Value (permuted)
Estrogen metabolism	2	7	29	*Ugt1a10*	*Sult1e*	29	5.29	<0.001
Glutathione and one‐carbon metabolism	3	24	65	*Mat1a, Cth, Gclc*		12	3.92	0.001
One‐carbon metabolism and related pathways	4	41	86	*Mat1a, Cth, Gclc, Gpx5*		10	3.81	0.001
Synthesis and degradation of ketone bodies	1	5	11	*Oxct1*		20	3.04	0.01
Pentose phosphate pathway	1	5	20	*Rpia*		20	3.04	0.029
Irinotecan pathway	1	6	13	*Ugt1a6*		17	2.72	0.011
Chemokine signaling pathway	7	155	199	*Ccl11, Cx3cr1, Adcy6*	*Ccl12, Gngt2, Ncf1, Crk*	5	2.55	0.014
Methylation	1	8	15	*Mat1a*		12	2.26	0.021
Phase I biontransformations, non P450	1	8	10	*Pon2*		12	2.26	0.055
Proteasome degradation	3	50	61	*Uba1, Psmd13*	*Psmb2*	6	2.22	0.042
Heme biosynthesis	1	9	22		*Uros*	11	2.08	0.023
Osteoblast	1	9	14	*Osteocalcin*		11	2.08	0.047

**Table 2:** KEGG pathways in which gene methylation was altered in the fetal liver in response to low maternal folate intake during pregnancy

KEGG pathway term	Pathway name	Total genes on pathway	Number of altered genes	Hypermethylated genes	Hypomethylated genes	*p*‐Value
mmu00140	Steroid hormone biosynthesis	45	4	*Ugt1a7c*	*Srd5a2, Sult1e1, Cyp3a13*	0.049

### 3.3 Integration of gene expression and DNA methylation changes in response to low maternal folate intake in the fetal liver

**Figure 3:**
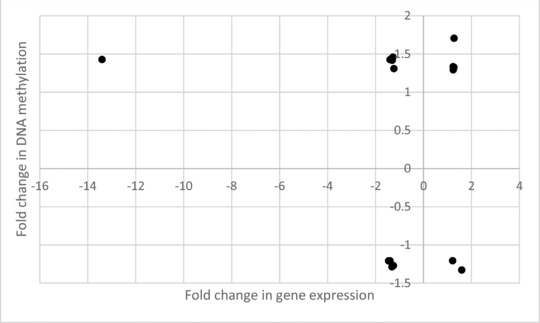
Scatterplot summarizing the direction and level of fold change in expression and in DNA methylation of genes with alterations in both parameters in fetal liver in response to maternal folate depletion.

In the fetal liver, 989 genes showed altered expression and 333 had altered methylation in response to maternal folate depletion but, of these, only 16 genes had both expression and methylation changes (Table 3 and Fig. 3). Furthermore, only seven of these genes displayed the expected inverse relationship between change in gene expression and change in promoter methylation (Table 3 and Fig. 3).

**Table 3:** Genes with changes in both gene expression and DNA methylation in the fetal liver in response to low maternal folate intake during pregnancy

Gene symbol	Gene name	Ensemble ID	Fold change in gene expression	Fold change in DNA methylation
*9330151L19RIK*	NA	NA	–1.28	1.46
*CCL11*	Chemokine (C–C motif) ligand 11	ENSMUSG00000020676	–1.40	1.42
*CYP3A13*	Cytochrome P450, family 3, subfamily a, polypeptide 13	ENSMUSG00000029727	1.59	–1.33
*DCBLD2*	Discoidin, CUB, and LCCL domain 2	ENSMUSG00000035107	–1.39	–1.21
*EBP*	Emopamil‐binding protein	ENSMUSG00000031168	1.28	1.70
*GLA*	Galactosidase alpha	ENSMUSG00000031266	1.23	1.33
*MAT1A*	Methionine adenosyltransferase I, alpha	ENSMUSG00000037798	1.27	1.33
*OXCT1*	Succinyl‐CoA:3‐oxoacid‐CoA transferase	ENSMUSG00000022186	–1.31	1.42
*PGM3*	Phosphoglucomutase 3,	ENSMUSG00000056131	1.24	1.29
*PHF14*	PHD finger protein 14	ENSMUSG00000029629	–1.32	–1.29
*PPP2R1A*	Protein phosphatase 2, regulatory subunit a, alpha	ENSMUSG00000007564	1.21	–1.21
*RECK*	Reversion‐inducing‐cysteine–rich protein with kazal motifs	ENSMUSG00000028476	–1.33	1.43
*SMPX*	Small‐muscle protein, X–Linked	ENSMUSG00000041476	–13.40	1.43
*SOX30*	SRY (sex‐determining region Y)–Box 30	ENSMUSG00000040489	–1.26	–1.27
*SRD5A2*	Steroid‐5‐alpha‐reductase	ENSMUSG00000038541	–1.46	–1.21
*TRAK1*	Trafficking protein, kinesin binding 1	ENSMUSG00000032536	–1.24	1.31

**Figure 4:**
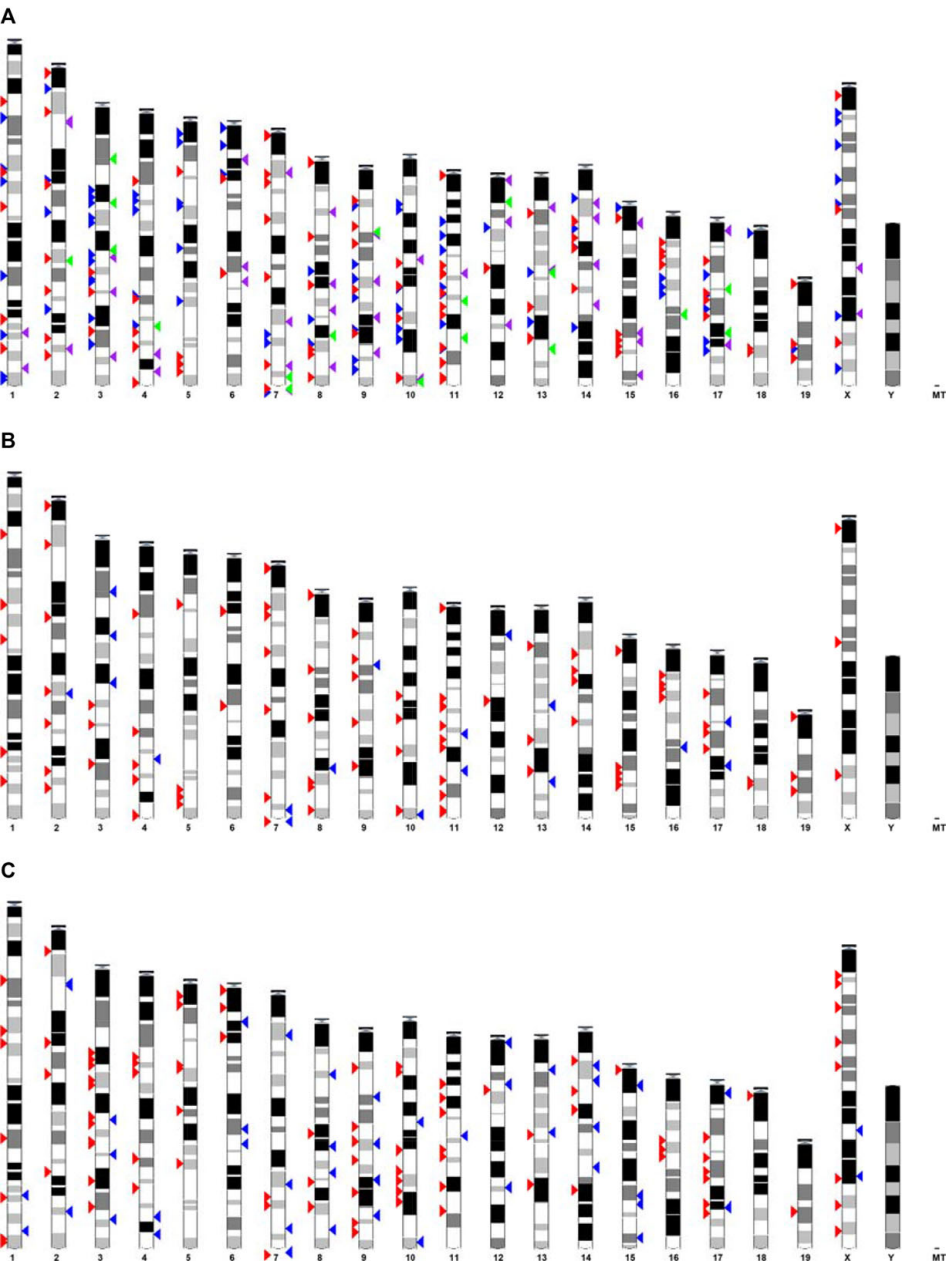
Chromosome locations for overrepresented regions of gene expression and DNA methylation changes in response to low maternal folate intake in the fetal liver. (A) Overrepresented regions of upregulated (red arrows), downregulated (blue arrows), hypermethylated (purple arrows), and hypomethylated (green arrows) genes. (B) Overrepresented regions of upregulated (red arrows) and hypomethylated (blue arrows) genes. (C) Overrepresented regions of downregulated (red arrows) and hypermethylated (blue arrows) genes.

PGE analysis was carried out to search for regions where the genome was overrepresented as a result of the maternal folate intervention. In response to low maternal folate intake, 124, 105, 50, and 18 genomic regions were found to be overrepresented as a result of upregulation, downregulation, hypermethylation, and hypomethylation, respectively, in the fetal liver (Supporting Information Tables 2–5). Mapping of these regions to chromosomal locations suggests that, while there were regions of the genome in which expression changes were not associated with methylation changes, in the majority of cases, gene expression and methylation changes occurred in neighboring regions (Fig. 4).

No common GO processes or KEGG pathways were identified that showed differential gene expression and differential gene methylation in the fetal liver response to maternal folate depletion. However, the Wikipathways one‐carbon metabolism and related pathways, methylation, and osteoblast showed both expression and methylation changes in the fetal liver from folate‐depleted mothers (Supporting Information Figs. 1–3).

## 4 Discussion

We hypothesized that DNA methylation may be an important mechanism involved in transcriptional changes in the fetal liver in response to low maternal folate intake during pregnancy, and therefore that gene expression changes would be concomitant with gene promoter methylation changes. While other regulatory mechanisms could contribute to the alterations in gene expression that we have observed (McKay et al., “Organ‐specific gene expression changes in the fetal liver and placenta in response to maternal folate depletion,” currently under review), we specifically hypothesized that DNA methylation changes may be an important mechanism through which folate intake influences transcriptional regulation because of the key role of folate as a methyl donor in one‐carbon metabolism and subsequent effect on the availability of SAM for the methylation of DNA 21, 41.

Using a genome‐wide array based approach, we identified 333 genes for which promoter methylation was altered in response to low maternal folate intake. We observed that the majority of the promoter regions that we interrogated appear to be protected from such epigenetic change. Since folate is a key input driving the one‐carbon cycle, we might have anticipated a bigger response in DNA methylation to maternal folate depletion. Indeed, Chen et al. reported altered methylation of 1034 genes in the liver of 21‐day‐old rat offspring in response to feeding a methyl donor deficient diet to their dams during pregnancy and lactation 42. However, only ten genes (*ABCA1, ANK3, BRWD1, CHRNB3, DCTN3, JAKMIP1, NCF1, PCDHB6, SLC36A3, TMEM188*) were found to have altered methylation in both Chen et al.’s study and the study described here. The observed difference in methylation response and very limited overlap in differentially methylated genes observed between the two studies may be due to important differences in dietary strategy. Chen et al. used a diet deficient in both folate and vitamin B_12_, while in our study dietary vitamin B_12_ was not altered and folate content was depleted, not deficient. In addition, the time point and animal models used may have influenced outcome measures, that is, we investigated methylation during murine fetal development, whereas Chen et al. analyzed livers of 21‐day‐old rat offspring. Our findings suggest that, when SAM availability is compromised through folate depletion, DNA methylation is relatively protected and that other methylation processes, for example, methylation of proteins and lipids, may be affected to a greater extent. This hypothesis is in accord with earlier mathematical modeling studies of folate‐mediated one‐carbon metabolism that found that DNA methylation reaction rate was relatively insensitive to changes in folate pool size 43. Here, we report changes in promoter methylation and in gene expression in pathways relating to one‐carbon metabolism and methylation in response to folate depletion, which may suggest that changes in these pathways could be direct feedback mechanisms that may protect against more widespread changes in DNA methylation patterns in response to folate depletion.

As lowered folate supply leads to reduced SAM availability, we expected to see more genes that were hypomethylated than hypermethyled in the fetal livers from folate‐depleted dams. However, we observed that nearly three times more gene promoters were hypermethylated as were hypomethylated in response to low maternal folate intake. This paradoxical finding is in accordance with other studies in rodents that reported that the intake of folate and/or other methyl donors was inversely associated with DNA methylation 44, 45. As the metabolic pathways of folate, choline, methionine, and vitamins B_6_ and B_12_ are closely interconnected 46, it is possible that the perturbation of one of these pathways may lead to compensatory changes in others. Indeed in support of this hypothesis, we observe that folate‐induced changes in methylation and gene expression were likely to have influenced one‐carbon metabolism and methylation pathways. Alternatively, the overall pattern of hypermethylation observed here may be a tissue specific response. Chung et al. reported tissue‐specific methylation patterns that differed between NTD‐affected and control fetuses; 5‐methylcytosine (5mC) content increased in kidney, but decreased in liver from wk 18 to 28 of fetal development in control but not NTD‐affected fetuses 47. Since low maternal serum folate concentrations were observed in NTD mothers, altered tissue‐specific methylation patterns may be associated with low folate status in NTD‐affected pregnancies 47. However, tissue‐specific methylation and maternal serum folate concentration were correlated in brain tissue only 47. It is plausible that other mechanisms, including disease‐related pathology, may be responsible for the observed tissue‐specific methylation patterns in NTD‐affected pregnancies.

When transcriptomic and methylomic datasets were integrated, we found just 16 genes that showed changes in both expression and promoter methylation. Moreover, only seven of these genes displayed the expected inverse association between change in expression and change in methylation. While nonsynonymous changes in gene expression and methylation have been reported in a number of integrated transcriptomic and methylomic studies 42, 48, 49, 50, we had anticipated a stronger correlation between promoter methylation and gene expression in the present study because of the direct influence of folate supply on the amount of SAM available for DNA methylation. Chen et al. observed simultaneous changes in expression and promoter methylation for 266 genes in the livers of 21‐day‐old rat offspring born to mothers fed a methyl donor deficient diet during pregnancy and lactation, but did not report the direction of methylation change 42. There was no overlap between genes with concomitant expression and methylation changes reported in Chen et al.’s study and the 16 concomitant genes reported here, which may be due to differences in study design discussed above.

It is plausible that the methylation status of a gene promoter may not influence the expression of that specific gene but could influence expression of a neighboring gene(s). For example, methylation changes within a *cis*‐acting enhancer 51 could influence the expression of a neighboring gene or multiple genes without affecting the gene nearest to the enhancer's location. To test this hypothesis we used PGE analysis to find regions of the genome enriched for changes in gene expression and methylation in response to maternal low folate intake. For most regions of methylation change, we identified gene expression changes in neighboring genomic regions. This suggests that regional methylation changes, rather than the more simplistic model of promoter methylation change, may regulate gene regulation, perhaps through *cis*‐acting enhancer mechanisms.

In this study, we used the Nimblegen MM8_RefSeq_promoter methylation array platform to measure genome‐wide methylation in fetal livers in response to maternal folate depletion during pregnancy. A strength of using this array‐based approach is that it enabled us to interrogate the methylation status of all known promoter regions across the genome. Given the importance of promoter regions in transcriptional regulation, and the evidence that promoter methylation is associated with gene silencing 52, we selected arrays that allowed quantification of methylation status of promoter regions (up to 2.6‐kb regions) of all well‐characterized mouse genes. However, a limitation of this approach is that we were unable to capture methylation changes occurring outside promoter regions that may have been altered in response to folate depletion and that could have caused changes in gene expression. There is growing evidence that DNA methylation at intragenic regions (reviewed in Kulis et al. 53) and CpG island shores 54 can influence gene expression patterns. Moreover, studies of cancer cells show that genes located in partially methylated domains 55 or long hypomethylated domains 56, 57 are more likely to be repressed, which adds further intricacies to the complex relationship between DNA methylation status and gene expression. Therefore, analysis of promoter regions only is likely to have restricted capacity to find synonymous associations between methylation patterns and gene expression. Finally, although the MeDIP method is robust in identifying regions of the genome with large‐scale methylation changes, it is less able to discern smaller regions of methylation changes, which may also be significant in terms of gene expression. While the integration of genome‐wide and epigenome‐wide data that we have undertaken here can offer insights into the effects of environmental exposures on gene expression and methylation, due to the limitations of the methodologies used to measure DNA methylation, the full complexity of the relationships between DNA methylation patterning and gene expression profiles remains to be uncovered. The application of technologies such as next‐generation sequencing to characterize and quantify changes in methylation may help to elucidate such relationships.

We found limited support for our hypothesis that altered promoter DNA methylation changes in gene expression in the fetal liver in response to low maternal folate intake. This suggests that other transcriptional regulatory mechanisms are more important in this context. Indeed, it is relevant to note that, although the presence of DNA methylation within gene promoters associates well with gene repression, the absence of DNA methylation in gene promoter regions does not always correlate with gene expression. For example, only approximately 50% of genes are expressed in particular cell types during early embryonic development when most of the genome is unmethylated 58, highlighting the importance of other regulatory mechanisms in determining patterns of gene expression. While we have not formally investigated other mechanisms of transcriptional control such as the role of enhancers, silencers, activators, or other epigenetic mechanisms, our reported transcriptomic data show that 7% of the genes that were differentially expressed in response to maternal folate depletion encoded transcription factors. These changes in transcription factors are likely to have been responsible for the altered expression of other multiple genes and, therefore, could explain some of the discordance observed between gene expression and DNA methylation observed in this study.

In summary, we observed that low maternal folate supply before mating and during pregnancy influences both expression and DNA methylation of multiple genes in the fetal liver. However, at an individual gene level, there was little overlap between genes showing altered expression and those showing altered promoter methylation. While the relationship between DNA methylation and gene expression changes in this model remains unclear, both are likely to be involved in modulating programming of the offspring in response to early‐life folate depletion and is revealed as adverse effects on offspring health in later life 59. Since epidemiological evidence links inadequate folate intake during pregnancy with increased risk of a range of adverse pregnancy and childhood health‐related outcomes in humans 4, 5, 6, 7, 8, 9, 10, 11, 12, 13, 14, 15, 16, findings from this animal model suggest that DNA methylation may be a mediating mechanism linking folate status and such outcomes. Folate inadequacy is common in women of child‐bearing age and has multiple public health implications, perhaps especially in those who are also obese 60. Further knowledge of the underlying molecular changes associated with inadequate folate intake during pregnancy may illuminate the causal pathways to disease and lead to potential biomarkers for disease screening programs.

*The authors have declared no conflict of interest*.

## Supporting information

As a service to our authors and readers, this journal provides supporting information supplied by the authors. Such materials are peer reviewed and may be re‐organized for online delivery, but are not copy‐edited or typeset. Technical support issues arising from supporting information (other than missing files) should be addressed to the authors.

**Supplementary Figure 1**. One carbon metabolism and related pathways modified from WikiPathways to highlight genes with altered expression levels and/or promoter methylation in response to maternal folate depletion in the fetal liver.
**Supplementary Figure 2**. Methylation pathway modified from WikiPathways to highlight genes with altered expression levels and/or promoter methylation in response to maternal folate depletion in the fetal liver.
**Supplementary Figure 3**. Osteoblast pathway modified from WikiPathways to highlight genes with altered expression levels and/or promoter methylation in response to maternal folate depletion in the fetal liver.

**Supplementary Table 1**. List of genes with differential methylation in response to low maternal folate intake in the fetal liver
**Supplementary Table 2**. Over represented genomic regions due to increased gene expression in reponse to low maternal folate intake in the fetal liver
**Supplementary Table 3**. Over represented genomic regions due to decreased gene expression in reponse to low maternal folate intake in the fetal liver
**Supplementary Table 4**. Over represented genomic regions due to hypermethylation of genes in reponse to low maternal folate intake in the fetal liver
**Supplementary Table 5**. Over represented genomic regions due to hypomethylation of genes in reponse to low maternal folate intake in the fetal liver
