# Supplementary material for: Gene promoter DNA methylation patterns have a limited role in orchestrating transcriptional changes in the fetal liver in response to maternal folate depletion during pregnancy
Source: Mol Nutr Food Res. 2016 Jun 6;60(9):2031–42. doi: 10.1002/mnfr.201600079 (PMC5031189; doi:10.1002/mnfr.201600079)
Supplement: Supplementary file 1 — Supplementary Figure 1. One carbon metabolism and related pathways modified from WikiPathways to highlight genes with altered expression levels and/or promoter methylation in response to maternal folate depletion in the fetal liver. Supplementary Figure 2. Methylation pathway modified from WikiPathways to highlight genes with altered expression levels and/or promoter methylation in response to maternal folate depletion in the fetal liver. Supplementary Figure 3. Osteoblast pathway modified from WikiPathways to highlight genes with altered expression levels and/or promoter methylation in response to maternal folate depletion in the fetal liver. [file MNFR-60-2031-s001.docx]

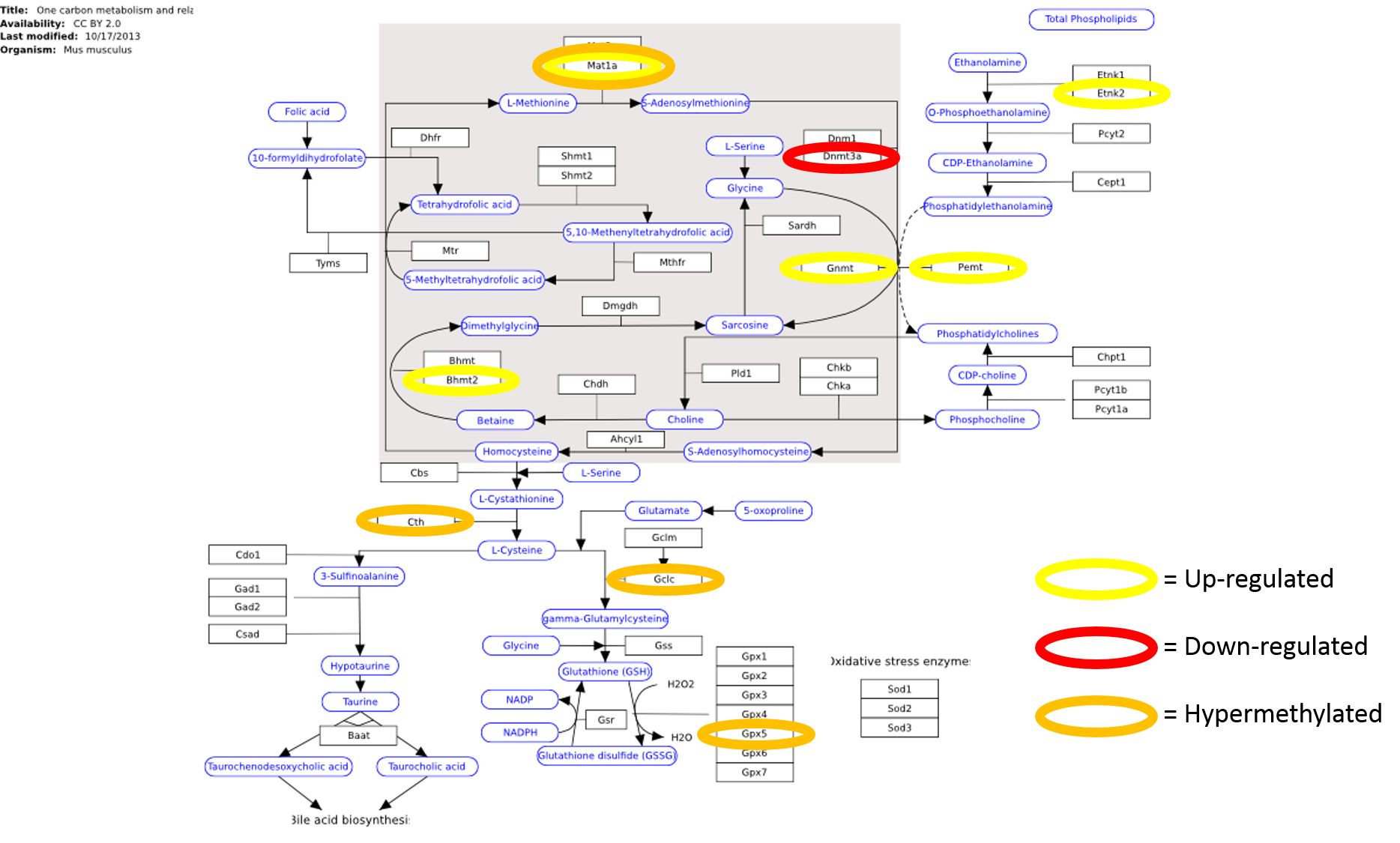


**Supplementary Figure 1.** One carbon metabolism and related pathways modified from WikiPathways to highlight genes with altered expression levels and/or promoter methylation in response to maternal folate depletion in the fetal liver.

**
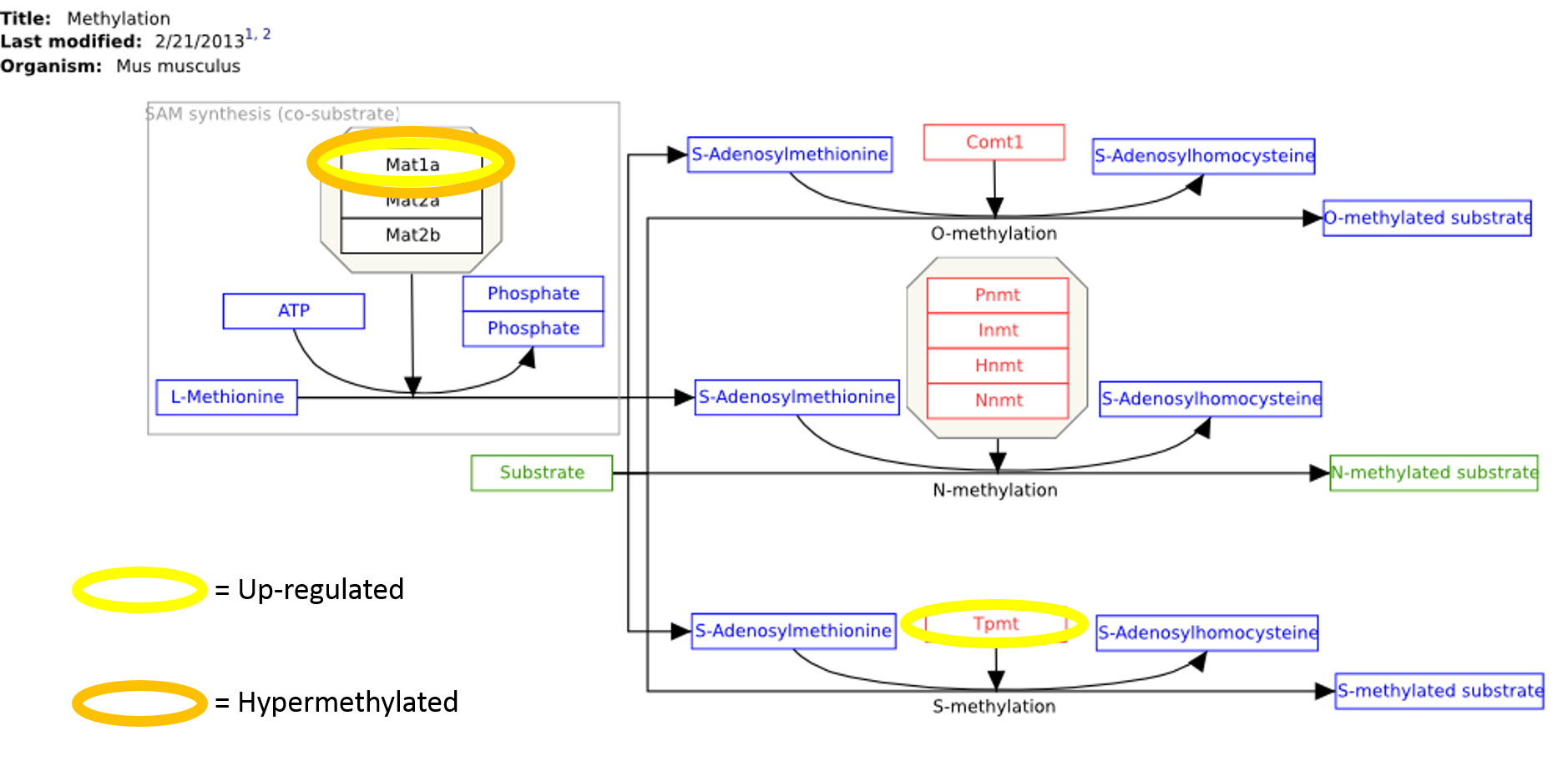
**

**Supplementary Figure 2.** Methylation pathway modified from WikiPathways to highlight genes with altered expression levels and/or promoter methylation in response to maternal folate depletion in the fetal liver.


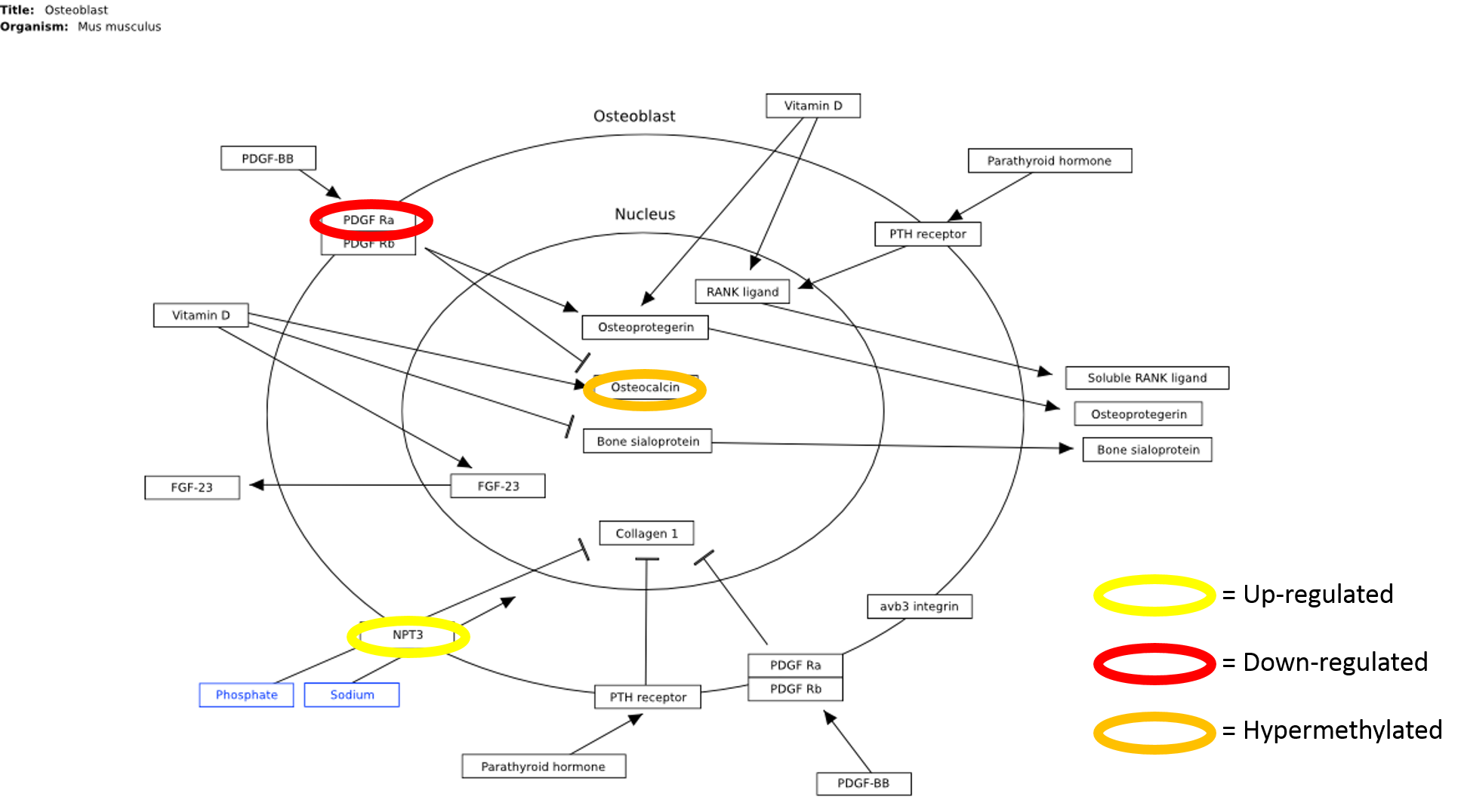


**Supplementary Figure 3.** Osteoblast pathway modified from WikiPathways to highlight genes with altered expression levels and/or promoter methylation in response to maternal folate depletion in the fetal liver.
